# Measurement of Systemic Mitochondrial Function in Advanced Primary Open-Angle Glaucoma and Leber Hereditary Optic Neuropathy

**DOI:** 10.1371/journal.pone.0140919

**Published:** 2015-10-23

**Authors:** Nicole J Van Bergen, Jonathan G. Crowston, Jamie E. Craig, Kathryn P. Burdon, Lisa S. Kearns, Shiwani Sharma, Alex W. Hewitt, David A. Mackey, Ian A. Trounce

**Affiliations:** 1 Centre for Eye Research Australia, University of Melbourne, Royal Victorian Eye and Ear Hospital, Melbourne, Australia; 2 Department of Ophthalmology, School of Medicine, Flinders University, Adelaide, Australia; 3 Centre for Ophthalmology and Visual Science, University of Western Australia, Perth, Australia; 4 Lions Eye Institute, Perth, Australia; Massachusetts Eye & Ear Infirmary, Harvard Medical School, UNITED STATES

## Abstract

Primary Open Angle Glaucoma (POAG) is a common neurodegenerative disease characterized by the selective and gradual loss of retinal ganglion cells (RGCs). Aging and increased intraocular pressure (IOP) are glaucoma risk factors; nevertheless patients deteriorate at all levels of IOP, implying other causative factors. Recent evidence presents mitochondrial oxidative phosphorylation (OXPHOS) complex-I impairments in POAG. Leber Hereditary Optic Neuropathy (LHON) patients suffer specific and rapid loss of RGCs, predominantly in young adult males, due to complex-I mutations in the mitochondrial genome. This study directly compares the degree of OXPHOS impairment in POAG and LHON patients, testing the hypothesis that the milder clinical disease in POAG is due to a milder complex-I impairment. To assess overall mitochondrial capacity, cells can be forced to produce ATP primarily from mitochondrial OXPHOS by switching the media carbon source to galactose. Under these conditions POAG lymphoblasts grew 1.47 times slower than controls, whilst LHON lymphoblasts demonstrated a greater degree of growth impairment (2.35 times slower). Complex-I enzyme specific activity was reduced by 18% in POAG lymphoblasts and by 29% in LHON lymphoblasts. We also assessed complex-I ATP synthesis, which was 19% decreased in POAG patients and 17% decreased in LHON patients. This study demonstrates both POAG and LHON lymphoblasts have impaired complex-I, and in the majority of aspects the functional defects in POAG were milder than LHON, which could reflect the milder disease development of POAG. This new evidence places POAG in the spectrum of mitochondrial optic neuropathies and raises the possibility for new therapeutic targets aimed at improving mitochondrial function.

## Introduction

Glaucoma is a neurodegenerative disease characterized by the selective and accelerated loss of retinal ganglion cells (RGCs). It has been spectulated that mitochondrial dysfunction contributes to Primary Open-Angle Glaucoma (POAG) [1–9]. RGCs are particularly sensitive to mitochondrial dysfunction, as exemplified by two diseases: Leber Hereditary Optic Neuropathy (LHON), caused by mitochondrial DNA (mtDNA)-linked defects in oxidative phosphorylation (OXPHOS) complex-I [10], and Autosomal Dominant Optic Atrophy (ADOA), caused in most cases by mutations in a nuclear gene encoding the mitochondrial fusion protein OPA1 [11–13]. In late-stage disease POAG and LHON share phenotypical similarities at the optic nerve head, and both suffer RGC loss [14–16]. A high density of mitochondria at the optic nerve head suggests a particular dependence on mitochondrial function in this location, which predisposes RGCs to metabolic failure when additional stresses such as age and increased intraocular pressure (IOP) occur [17].

Raised IOP is a major risk factor for POAG [18] and lowering IOP can slow progression [19], nevertheless patients continue to deteriorate despite IOP lowering [20] which implies that other risk factors contribute to POAG. There is emerging evidence of mitochondrial impairment in glaucoma [5,21]. Functional studies from advanced POAG patients revealed a decreased total mitochondrial respiratory function in lymphocytes [22] and trabecular meshwork cells [23] that was likely due to a complex-I impairment [24]. Interestingly, patients experiencing high IOP for many years without optic nerve degeneration had enhanced mitochondrial function which potentially protects their optic nerve against IOP stress [25]. Here we report a validation study of our previous report [24] using a cohort of advanced POAG and control patients collected from the Australian and New Zealand Registry of Advanced Glaucoma (ANZRAG) [26–31], a well-characterised advanced glaucoma and control patient cohort. Furthermore we compare the degree of complex-I impairment in advanced POAG patients to that of LHON patients with advanced vision loss to correlate the degree of mitochondrial impairment to that of disease development.

## Materials and Methods

### Patient selection and lymphoblast line generation

For POAG cases, patients and controls from the ANZRAG patient cohort were examined by an experienced ophthalmologist [27]. The ANZRAG cohort is the largest cohort of advanced POAG patients and controls recruited across Australian and New Zealand in order to identify new clinical and genetic risk factors for developing the worst outcomes in glaucoma. Detailed patient information is collected, including clinical examination, family history and environmental risk profile. In the ANZRAC cohort patients with advanced POAG fulfilled the following criteria in the worst eye: visual field loss related to glaucoma with at least two out of the four central squares having a pattern standard deviation <0.5% on a Humphrey 24–2 field or a mean deviation of <−22 dB, or in the absence of field testing, loss of central acuity related to glaucoma was tested using a Snellen visual acuity chart with either pinhole or full refractive error correction [27]. Subjects were also required to have evidence of glaucomatous optic disc changes (even if mild) for the better seeing eye. Detailed clinical assessment was performed and documented as previously described for the ANZRAG recruitment [27].

For this study advanced POAG patients and controls were carefully selected from the larger ANZRAG cohort by experienced ophthalmologists (AWH and JEC) and geneticists (KPB and SS). Exclusion criterion for this cohort included; the presence of any other ocular, systemic, chronic or neurological diseases other than POAG-related optic nerve damage, the presence of any glaucoma types other than POAG and the presence of cancer. Stringent exclusion criteria were maintained to reduce the risks of chronic diseases impacting on mitochondrial function. Controls were matched from the same patient cohort for age and gender. Patient summary information for POAG and age-matched controls can be found in Table 1.

**Table 1:** POAG patient demographics.

	Controls (Cont1)	POAG
**Number**	20	15
**Age (years)**	79 ± 7	80 ± 7
**Gender**	11 F, 9 M	7 F, 8 M
**Smokers**	7/20	3/15
**Diabetes**	1/20	3/15
**Hypertension**	9/20	8/15
**Thyroid problems**	3/20	1/15
**Arthrosclerosis**	5/20	8/15
**Steroid medication**	9/20	5/15
**Migraine or headache**	4/20	3/15
**First-degree relative with POAG**	n/a	4/15
**Disease duration (years)**	n/a	17 ± 9
**Highest IOP**	15 ± 1	28 ± 8
**Mean Deviation OD / OS**	n/a	-20 ± 8 / -18 ± 9
**Central corneal thickness OD / OS**	542 ± 28 / 541 ± 46	505 ± 49 / 505 ± 46
**Cup-to-disc ratio OD / OS**	n/a	0.9 ± 0.1 / 0.9 ± 0.1

Control and POAG lymphoblasts were matched for age, sex and race. OD, right eye; OS, left eye. Continuous variables are presented as means ± standard deviation.

LHON patients were recruited through the Royal Victorian Eye and Ear Hospital (RVEEH) clinical genetics unit and patients known to carry the complex-I 11778 G>A mutation were seen by their consulting ophthalmologist. Patients were recruited with acute vision loss above the age of 20 years according to published criteria [32,33]. Visual acuity in affected patients ranged from 1/24 in the better eye through to detection of hand motion in the worst eye. Genotype of the transformed cell lines derived from LHON patients was confirmed by Sanger sequencing. The LHON cohort had an average age of 30 ± 15 years, and comprised of 4 males and 2 females. Controls for the LHON cohort (Cont2) were 58 ± 8 years and comprised of 5 males and 1 female. Patient summary information for LHON and age-matched controls can be found in Table 2.

**Table 2:** LHON patient demographics.

	Controls (Cont2)	LHON
**Number**	6	6
**Age (years)**	58 ± 8	30 ± 15
**Gender**	5M, 1F	4M, 2F
**Visual acuity**	Normal vision	1/24 –HM

Control and LHON lymphoblasts were matched for age, sex and race. Continuous variables are presented as means ± standard deviation. HM: detection of hand motion in the worst eye.

Ethics approval was obtained from the Southern Adelaide and Flinders University Clinical Research Ethics Committee for collection of the POAG cohort, and from the RVEEH Clinical Research Committee for collection of the LHON cohort. The study was conducted in accordance with the revised Declaration of Helsinki and following the National Health and Medical Research Council (Australia) statement of ethical conduct in research involving humans. Written informed consent was provided by all participants.

Epstein Barr Virus (EBV)-transformed lymphoblast lines were generated from blood samples collected in EDTA blood collection tubes (Greiner BioOne) and stored at room temperature prior to transformation. Lymphocytes were transformed using EBV as previously described [34]. Control lymphoblast lines were age-and gender-matched to the POAG cohort and were generated at the Department of Genetic Medicine, Women’s and Children’s Hospital, North Adelaide, Australia. Lymphoblast lines for the LHON and age- and gender-matched control cohort were generated at the Centre for Eye Research Australia.

### Lymphoblast culture

Lymphoblasts were maintained in RPMI-1640 media containing 12 mM glucose, 15% heat-inactivated fetal bovine serum (FBS), 2.05 mmol/L L-glutamine, 100 units/mL penicillin, and 100 μg/mL streptomycin. Lymphoblast lines were also grown in glucose-free galactose RPMI-1640 media, containing 5 mM galactose, 4.5 mM sodium pyruvate, 15% dialyzed heat-inactivated FBS, 2.05 mmol/L L-glutamine, 100 units/mL penicillin, and 100 μg/mL streptomycin. All lymphoblast lines were cultured as described in 125 cm^2^ tissue culture flasks (Greiner Bio-one, Germany) and incubated at 37°C, 5% CO_2_ in a humidified incubator. For all experiments (ATP production, respiration, lymphoblast pellet harvesting for OXPHOS enzymology) lymphoblast lines were seeded at 2x10^5^ cells/ml in fresh RPMI media 3 days prior to performing experiments or lymphoblast harvesting to ensure equal lymphoblast proliferation rates between groups. All lymphoblast lines were randomised and experiments were performed in large batches to minimise inter-experiment variation, and analysis was performed blinded to sample identity.

### PCR genotyping for LHON mutation

Presence of the LHON 11778 G>A mutation was confirmed by PCR and Sanger sequencing. DNA was extracted from lymphoblasts using Qiagen genomic DNA extraction kit according to manufacturer’s protocols. PCR amplification was performed using Taq DNA polymerase (Invitrogen) and the following forward primer (5’-3’—CCC ACC TTG GCT ATC ATC) and reverse primer (5’-3’—GGT AAG GCG AGG TTA GCG) for 25 cycles of 94°C for 30 sec, 51°C for 30 sec and 72°C for 60 sec. PCR product size was confirmed by agarose gel electrophoresis, residual primers removed with a PCR clean-up kit (Qiagen) and PCR fragments sequenced by Sanger sequencing at the Australian Genomic Research Facility (Melbourne, Australia) using the forward primer. Sequences were aligned to the revised Cambridge mitochondrial DNA sequence (GenBank sequence NC_012920).

### Lymphoblast proliferation in galactose media

Growing cells in media in which glucose is removed and galactose is provided as a carbon source is a commonly used screening test for mitochondrial dysfunction. Because cells grown in galactose rely on OXPHOS to synthesise ATP, cells with mitochondrial impairments have slower proliferation rates and increased population doubling times [35]. Lymphoblast viability and lymphoblast number were assessed with the trypan blue exclusion assay. Lymphoblasts were cultured in either glucose or galactose media at an initial cell number of 2x10^5^ cells/ml. At each timepoint between 0–9 days an aliquot of resuspended lymphoblasts was stained with 0.04% (w/v) trypan blue and counted using an automated hemocytometer (Countess automated cell counter, Invitrogen). Doubling time, the time taken for the lymphoblast population to double (days) was calculated during the exponential phase of proliferation. For glucose-grown lymphoblasts this was days 2–4, and for galactose-grown lymphoblasts this was days 1–6. These rates were automatically calculated using Prism 5.01 software (GraphPad Software Inc.). The ‘exponential growth’ nonlinear regression model and the fitting method of ‘least squares (ordinary fit)’ from Prism was used to automatically calculate doubling time.

### OXPHOS-specific activity assays

All assays were performed using a Cary 300 Bio (Varian, CA, USA) single beam spectrophotometer. Frozen lymphoblast pellets from cultures harvested during exponential proliferation were thawed from -80°C and resuspended in 100μl of mannitol buffer (225mM mannitol, 75mM sucrose, 10mM Tris-Cl, 0.1mM EDTA, pH 7.4 with KOH), and sonicated briefly on ice (Milsonix homogeniser, 4 pulses at 0.04 V, power setting 1.5). Prepared samples were assayed in quartz cuvettes. Normalisation of OXPHOS-specific activity to the activity of the Kreb’s cycle enzyme citrate synthase allowed for correction for mitochondrial density [36,37].

Complex-I (NADH: ubiquinone oxoreductase, EC 1.6.5.3) activity was measured by monitoring oxidation of NADH using the extinction coefficient of 6.22 mM^-1^.cm^-1^. Briefly, 500μg lymphoblast lysate was added to the complex-I reaction mixture (100μM NADH, 100μM decylubiquinone, 3.75mg/ml BSA) in phosphate buffer (50mM KH_2_PO_4_/ 50mM K_2_HPO_4_, pH 7.5) and oxidation of NADH was monitored for 5 minutes at 340nm and 37°C. The specific complex-I activity was calculated by subtracting the rotenone insensitive activity from the total NADH ubiquinone oxidoreductase activity by running parallel reactions with the complex-I inhibitor rotenone (12.5μM). Rotenone insensitive activity usually accounted for less than 10% of the overall activity. Complex-IV (ferrocytochrome c:oxygen oxidoreductase, EC 1.9.3.1) activity was measured as described previously [34]. 400μg lymphoblast lysate was added to assay medium, and the rate of oxidation of ferrocytochrome c was determined at 550nm and 30°C. Complex-IV specific activity was calculated using the extinction coefficient of 27.2 mM^-1^.cm^-1^. Citrate synthase (EC 4.1.3.7) activity was measured as described previously [34]. Briefly 200μg lymphoblast lysate was added to assay medium and monitoring DTNB reduction monitored at 412 nm and 30°C. Citrate synthase-specific activity was calculated using the extinction coefficient of 13.6 mM^-1^.cm^-1^.

### Mitochondrial ATP synthesis

Maximal mitochondrial ATP synthesis for complex-I (glutamate + malate) and complex-II (succinate + rotenone) can be measured in permeabilised cells in the presence of excess ADP to determine mitochondrial efficiency. ATP synthesis rates were measured by using a luciferin/luciferase assay as previously described [38–40] with some modifications [34]. The measurement of mitochondrial ATP synthesis was performed in lymphoblasts grown in RPMI with 12 mM glucose and 15% heat inactivated FBS for 72 hours. The rate of ATP synthesis was linear and dependent on lymphoblast density [34], and oligomycin inhibited >95% of mitochondrial ATP synthesis.

### NAD^+^/NADH ratio

Lymphoblasts were seeded at 1x10^6^ cells/ml in standard RPMI (2mg/ml glucose) with 15% heat-inactivated fetal calf serum for 48 hours prior to harvesting lymphoblasts for analysis. At the time of harvesting, lymphoblasts were counted, resuspended at 1x10^6^ cells/ml in PBS and 700μl transferred to a new tube. An equal volume of bicarbonate buffer (100mM sodium carbonate, 20mM sodium bicarbonate, 10mM nicotinamide, 0.05% Triton-X 100, approximate pH 10–11) + 1% dodecyltrimethylammonium bromide (DTAB) was added, lymphoblasts were gently mixed to lyse and preserve the dinucleotides, then snap-frozen at -80°C for future analysis. NAD^+^ and NADH levels were measured using the NAD/NADH-Glo assay (Promega) in two separate reactions designed to detect specifically either NAD^+^ or NADH, according to Part 5 of the manafacturers protocol. Data was compared to a standard curve of NAD^+^ (Sigma, N7004), and from this the NAD^+^ and NADH amounts were calculated as well as the NAD^+^ to NADH ratio.

### Lactate production

Mitochondrial impairment can lead to elevated lactate levels [41], which is a common, albeit a non-specific marker of mitochondrial disease [42]. Lymphoblasts were grown in GUP media (G = glucose raised to 4mg/ml from the standard RPMI level of 2mg/ml, U = uridine at 50 μg/ml, P = pyruvate at 1mM) in phenol-red free RPMI (Invitrogen) and 5% heat-inactivated FBS (Invitrogen). Phenol-red free media was used as phenol interferes with lactate measurements, and serum was reduced to 5% to minimise interference from lactate present in serum. Briefly, lymphoblasts were seeded at 1x10^6^ cells/ml in GUP media, incubated for 48 hours then pelleted prior to a media sample being removed for lactate measurements. Lactate was quantified using a colourmetric assay (Sigma, MAK065) according to manufacturer’s protocols. Lactate was measured in samples diluted 1:40 in lactate dilution buffer, and was compared to a lactate standard curve. A media-only sample served as a blank and was subtracted from all other values.

### Statistical analysis

Each experiment was performed with at least 3 biological replicates per cell line and the average of these used for data analysis. Data normality was assessed with D'Agostino-Pearson normality test. For normally distributed data (OXPHOS, NAD^+^ and NADH) a two-sided unpaired Students test was performed and data was presented as mean values ± standard deviation (SD). Non-normally distributed data (doubling time, ATP synthesis, NAD^+^/NADH and lactate) was assessed by Mann-Whitney test and data was presented as median ± interquartile range (IQR). The accepted level of significance in all cases was P<0.05. All statistical analyses were performed with commercially available software (GraphPad Prism version 5.01 for Windows, GraphPad Software, San Diego California USA)

## Results

### Metabolic profiling of POAG and LHON lymphoblasts

**Fig 1:**
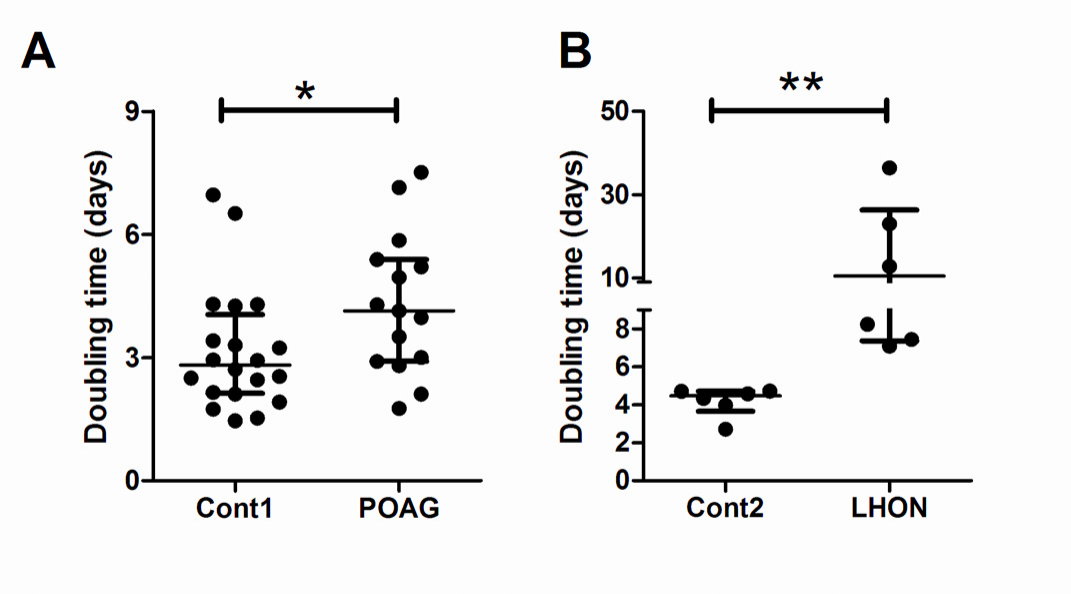
Impaired population doubling time in POAG and LHON lymphoblasts when forced to rely on OXPHOS. Control, POAG and LHON lymphoblasts were grown either in glucose media or galactose media, which forces cells to rely on OXPHOS for proliferation and survival. Proliferation curves were used to calculate population doubling time. A) POAG and B) LHON lymphoblasts had significantly longer population doubling time in galactose media when compared to controls. Data is median (IQR), Mann-Whitney test, n = 20 cont1, n = 15 POAG, n = 6 cont2 and n = 6 LHON, * = p< 0.05, ** = p< 0.01. Glucose media (RPMI-1640 media containing 2mg/ml glucose, 15% heat-inactivated serum, 2.05mM l-glutamine); Galactose media (glucose-free RPMI-1640 containing 5mM galactose, 4.5mM sodium pyruvate, 15% dialysed heat inactivated FBS, 2.05mM l-glutamine).

Lymphoblasts exposed to glucose-free galactose media are forced to generate ATP through OXPHOS. This is a commonly used assay to screen for mitochondrial impairments [35]. Impaired population doubling time was observed in both POAG and LHON lymphoblasts when they were forced to grow in galactose media. Population doubling time was significantly higher in POAG lymphoblasts [median (IQR): 4.14 (2.90–5.39) days] compared to age-matched controls [median (IQR): 2.82 (2.12–4.04) days; Mann-Whitney test, p = 0.040; Fig 1A] where POAG lymphoblasts grew 1.47 times slower than controls. Likewise, population doubling time was significantly higher in LHON lymphoblasts [median (IQR): 10.51 (7.36–26.35) days] than for age-matched controls [median (IQR): 4.47 (3.66–4.72) days; Mann-Whitney test, p = 0.002; Fig 1B, Table 3] where LHON lymphoblasts grew 2.35 times slower than controls. The impairment in lymphoblast population doubling time was therefore greater in LHON lymphoblasts compared to POAG lymphoblasts.

**Table 3:** Summary of major findings.

	Galactose proliferation	OXPHOS enzymology		ATP synthesis		Redox status
	Doubling time (dT)	Complex-I: CS	Complex-IV: CS	Complex-I	Complex-II	NAD^+^ /NADH
Cont1	2.82 (2.12–4.04)	0.45 ± 0.09	0.42 ± 0.10	0.43 (0.37–0.55)	0.23 (0.19–0.32)	4.36 (4.21–5.29)
POAG	4.14 (2.90–5.39)	0.37 ± 0.12	0.43 ± 0.11	0.35 (0.30–0.37)	0.19 (0.16–0.22)	4.72 (4.36–5.06)
Difference	1.47 times slower	18%	na	19%	17%	na
p-value	0.040 **#**	0.032 *	ns *	0.019 **#**	0.020 **#**	ns **#**
Cont2	4.47 (3.66–4.72)	0.65 ± 0.06	0.30 ± 0.10	0.35 (0.30–0.56)	0.18 (0.13–0.30)	4.66 (4.28–5.64)
LHON	10.51 (7.36–26.35)	0.47 ± 0.11	0.23 ± 0.04	0.29 (0.23–0.31)	0.15 (0.10–0.23)	3.61 (2.95–3.86)
Difference	2.35 times slower	29%	na	17%	na	23%
p-value	0.002 **#**	0.005 *	ns *	0.030 **#**	ns **#**	0.004 **#**

Statistical analysis: #Mann-Whitney test, data is median (interquartile range; IQR)

* Students t-test, data is mean ± standard deviation (SD).

### Impaired OXPHOS complex-I activity in POAG and LHON lymphoblasts

**Fig 2:**
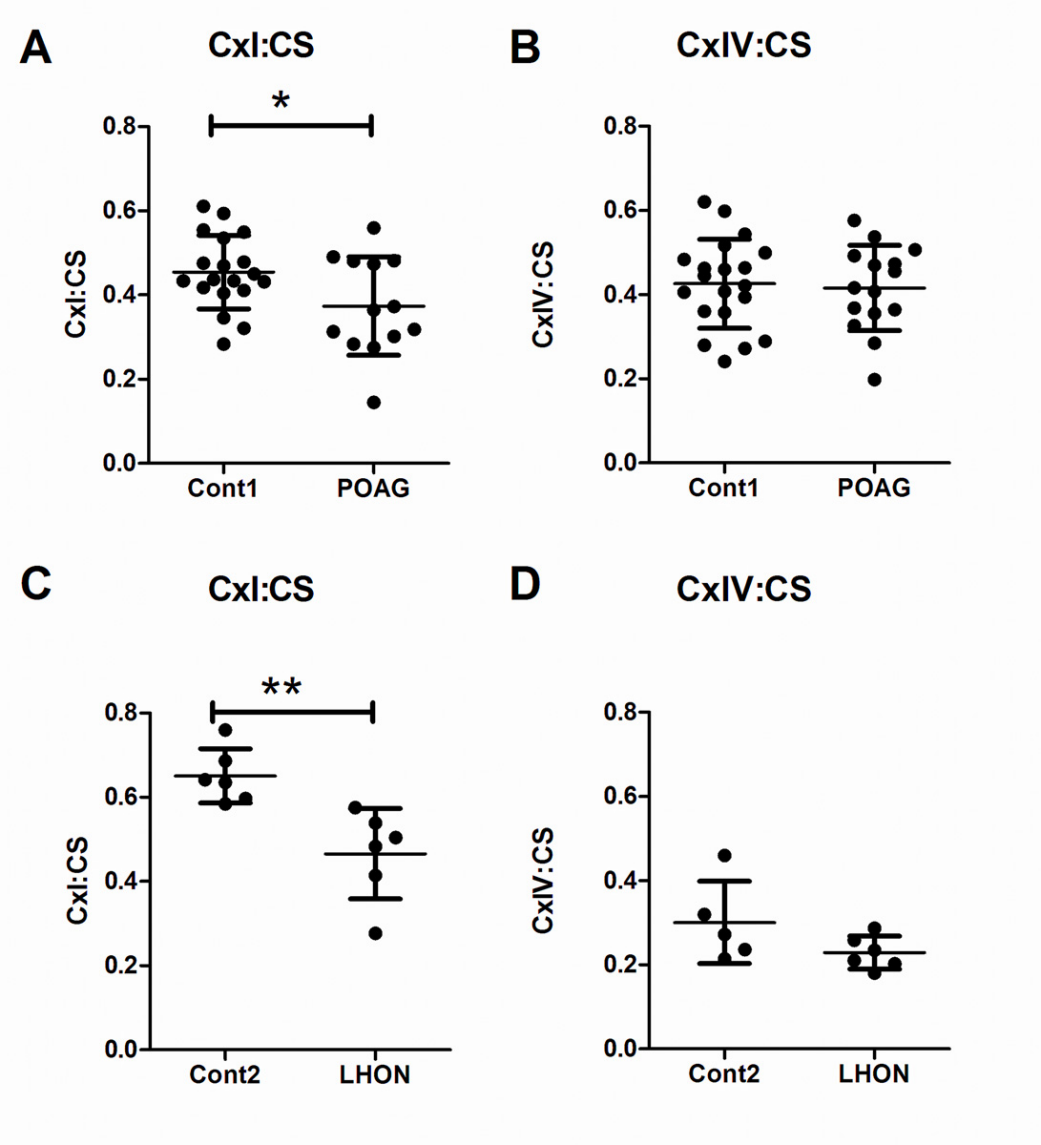
Impaired OXPHOS complex-I in POAG and LHON lymphoblasts. There was a significant decrease in complex-I specific activity in A) POAG and C) LHON lymphoblasts when specific activities were ratioed to that of the Kreb’s cycle enzyme citrate synthase. There was no change in the specific activity of Complex-IV for B) POAG or D) LHON lymphoblasts when ratioed to citrate synthase. Data is mean ± SD, students t-test, n = 20 cont1, n = 15 POAG, n = 6 cont2 and n = 6 LHON, * = p< 0.05, ** = p< 0.01.

To investigate the basis of the proliferation impairment in galactose media, we analysed OXPHOS enzymatic function in our POAG and LHON patient cohorts. Batches of lymphoblast pellets were used to measure the specific activity of complexes-I and -IV, and enzyme rates were ratioed to citrate synthase. Complex-I (rotenone sensitive) specific activity was significantly decreased in POAG lymphoblasts [mean ± SD: 0.37 ± 0.12 nmol/min/mg protein] versus age-matched controls [mean ± SD: 0.45 ± 0.09 nmol/min/mg protein; student’s t-test, p = 0.032; Fig 2A] which represented an 18% decrease in specific activity. Complex-IV activity remained unchanged in POAG lymphoblasts [mean ± SD: 0.43 ± 0.11 nmol/min/mg protein] compared to age-matched controls [mean ± SD: 0.42 ± 0.10 nmol/min/mg protein; student’s t-test, p = 0.769; Fig 2B]. When the same enzymes were measured in LHON lymphoblasts we observed the expected complex-I impairment [43] in LHON lymphoblasts [mean ± SD: 0.47 ± 0.11 nmol/min/mg protein] versus age-matched controls [mean ± SD: 0.65 ± 0.06 nmol/min/mg protein; students t-test, p = 0.005; Fig 2C] which represented a 29% decrease in specific activity. This was in the absence of any change in complex-IV activity with LHON [mean ± SD: 0.23 ± 0.04 nmol/min/mg protein] and age-matched controls having similar activities [mean ± SD: 0.30 ± 0.10 nmol/min/mg protein; student’s t-test, p = 0.130; Fig 2D]. The degree of complex-I activity impairment was greater in LHON lymphoblasts (29% decreased) than in POAG lymphoblasts (18% decreased).

### Impaired complex-I ATP synthesis in POAG and LHON lymphoblasts

**Fig 3:**
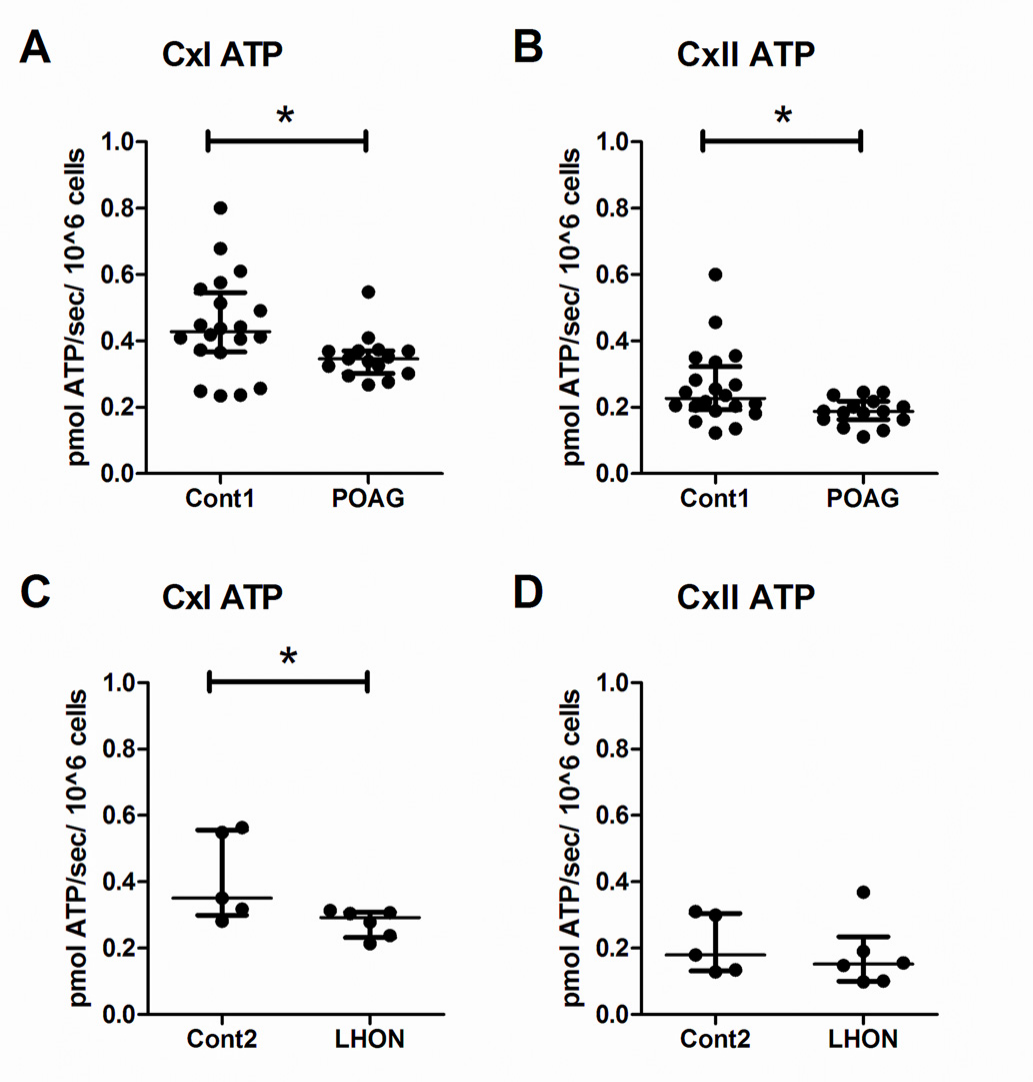
Impaired ATP synthesis in POAG and LHON lymphoblasts. Digitonin-permeabilised lymphoblasts from POAG, LHON and control patients were used to measure maximal ATP synthesis by mitochondrial respiration. There was a significant decrease in complex-I (glutamate + malate) driven ATP synthesis in both A) POAG and C) LHON lymphoblasts. There was a significant decrease in complex-II (succinate + rotenone) driven ATP synthesis in B) POAG lymphoblasts, but not in D) LHON lymphoblasts. Data is median (IQR), Mann-Whitney test, n = 20 cont1, n = 15 POAG, n = 6 cont2 and n = 6 LHON, * = p< 0.05.

We previously identified a complex-I defect in POAG [24]. In this replication cohort we measured the rate of mitochondrial ATP synthesis in digitonin-permeabilised lymphoblasts provided with complex-I substrates (glutamate + malate) or complex-II substrate (succinate + rotenone) in the presence of ADP. We found a significant decrease in the complex-I ATP synthesis rates in POAG lymphoblasts [median (IQR): 0.35 (0.30–0.37) pmol ATP/sec/10^6^ cells] versus age-matched controls [median (IQR): 0.43 (0.37–0.55) pmol ATP/sec/10^6^ cells; Mann-Whitney test, p = 0.019; Fig 3A], which represented a 19% decrease in complex-I ATP synthesis in POAG lymphoblasts. Similarly, there was a significant decrease in complex-II-driven ATP synthesis in POAG lymphoblasts [median (IQR): 0.19 (0.16–0.22) pmol ATP/sec/10^6^ cells] versus age-matched controls [median (IQR): 0.23 (0.19–0.32) pmol ATP/sec/10^6^ cells; Mann-Whitney test, p = 0.020; Fig 3B], which represented a 17% decrease in complex-II ATP synthesis in POAG lymphoblasts. The rates of complex-I driven ATP synthesis were also significantly decreased in LHON lymphoblasts [median (IQR): 0.29 (0.23–0.31) pmol ATP/sec/10^6^ cells] compared to age-matched controls [median (IQR): 0.35 (0.30–0.56) pmol ATP/sec/10^6^ cells; Mann-Whitney test, p = 0.030; Fig 3C], which represented a 17% decrease in complex-I ATP synthesis in LHON lymphoblasts. However there was no decrease in complex-II driven ATP synthesis in LHON lymphoblasts [median (IQR): 0.15 (0.10–0.23) pmol ATP/sec/10^6^ cells] compared to age-matched controls [median (IQR): 0.18 (0.13–0.30); Mann-Whitney test, p = 0.662; Fig 3D]. We found an equal decrease in complex-I-driven ATP synthesis in POAG and LHON lymphoblasts, and a decrease in complex-II ATP synthesis only in POAG lymphoblasts.

### Impaired NAD^+^ ratio in LHON

**Fig 4:**
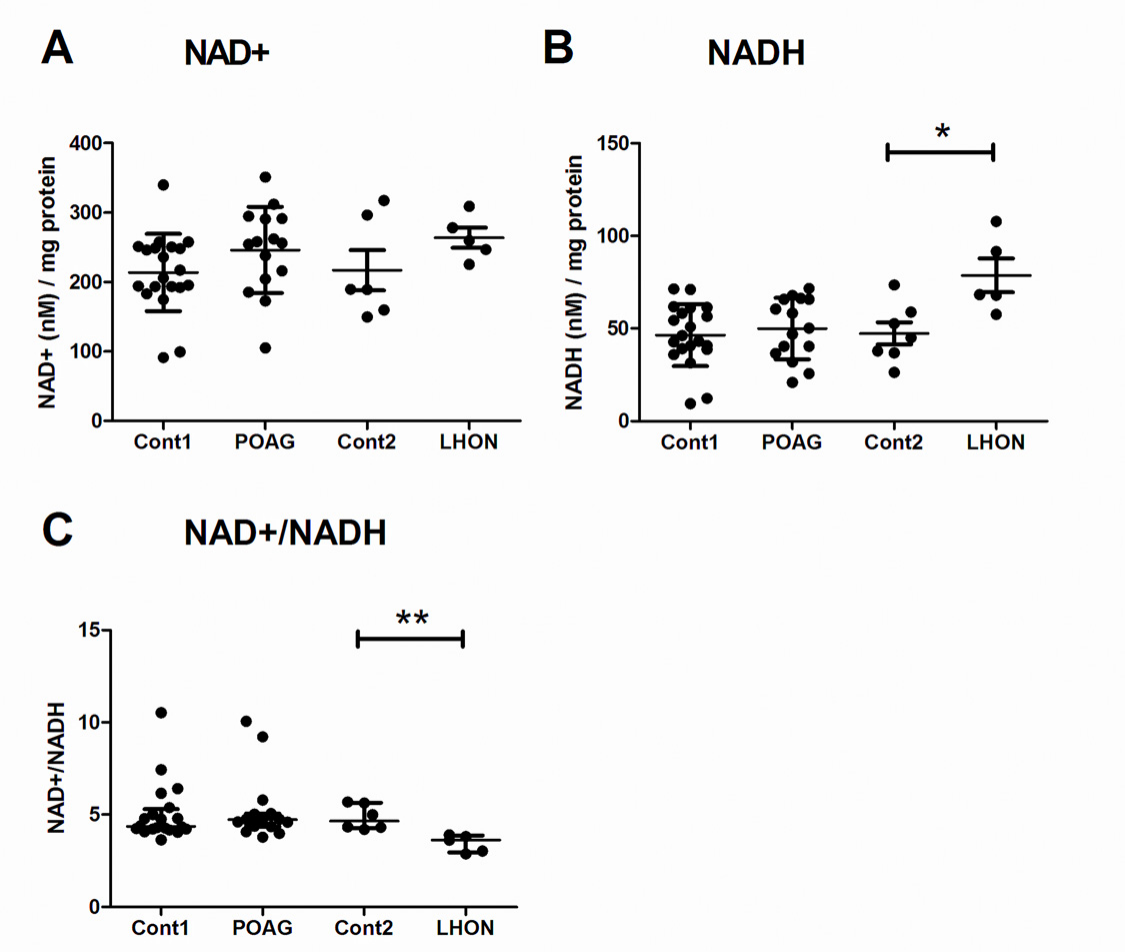
Decreased NAD^+^/NADH redox balance in LHON lymphoblasts. Lymphoblasts were grown in regular RPMI media for 48 hours prior to lymphoblasts being harvested to measure NAD^+^ and NADH levels. There was no significant difference in A) NAD^+^ levels between any groups; however there was a significant increase in B) NADH levels in LHON patients leading to C) a significant decrease in the NAD^+^/NADH ratio in LHON lymphoblasts. There was no difference between any of these measured parameters in POAG lymphoblasts, and cellular protein content was similar between groups (data not shown). For NAD^+^ and NADH data is mean ± SD, students t-test, and for NAD^+^/NADH data is median (IQR), Mann-Whitney test, n = 20 cont1, n = 15 POAG, n = 6 cont2 and n = 6 LHON, * = p< 0.05, ** = p< 0.01.

Complex-I converts NADH to NAD^+^ as part of electron transfer during ATP synthesis and levels are tightly regulated within a cell (commonly referred to as the NAD^+^/NADH ratio) which reflects the cellular redox state. When complex-I is severely impaired NADH accumulates and the ratio of NAD^+^/NADH decreases, leading to a reduced state within the mitochondrial matrix. When the cellular levels of NAD^+^ and NADH were measured in both POAG and LHON lymphoblasts we saw no difference in total NAD^+^ levels (Fig 4A). Total NAD^+^ in POAG lymphoblasts [mean ± SD: 246 ± 62 nM NAD^+^/mg protein] was no different to controls [mean ± SD: 214 ± 56 nM NAD^+^/mg protein; student’s t-test, p = 0.65; Fig 4A], likewise there was no difference in LHON [mean ± SD: 263 ± 32 nM NAD^+^/mg protein] and age-matched controls [mean ± SD: 217 ± 72 nM NAD^+^/mg protein; student’s t-test, p = 0.14; Fig 4A]. However total NADH was significantly higher in LHON lymphoblasts [mean ± SD: 79 ± 20 nM NADH/mg protein] versus age-matched controls [mean ± SD: 47 ± 16 nM NADH/mg protein; student’s t-test, p = 0.01; Fig 4B]. This was in the absence of any changes in total NADH levels in POAG [mean ± SD: 50 ± 17 nM NADH/mg protein] compared to age-matched controls [mean ± SD: 46 ± 17 nM NADH/mg protein; student’s t-test, p = 0.53; Fig 4B]. The higher total NADH in LHON lymphoblasts also corresponded to a significant decrease in the NAD^+^/NADH in LHON lymphoblasts [median (IQR): 3.61 (2.95–3.86)] compared to age-matched controls [median (IQR): 4.66 (4.28–5.64); Mann-Whitney test, p = 0.004; Fig 4C] which represented a 23% decrease. NAD^+^/NADH remained unchanged in POAG lymphoblasts [median (IQR): 4.72 (4.36–5.06)] versus age-matched controls [median (IQR): 4.36 (IQR 4.21–5.29; Mann-Whitney test, p = 0.68; Fig 4C]. This implies that LHON lymphoblasts with a more severe mitochondrial defect (complex-I enzyme activity and growth under galactose) have imbalanced redox levels in this cellular model. The POAG lymphoblast mitochondrial defect is more modest than in LHON lymphoblasts and is not severe enough to alter the redox status of the cells.

### Lactate production

**Fig 5:**
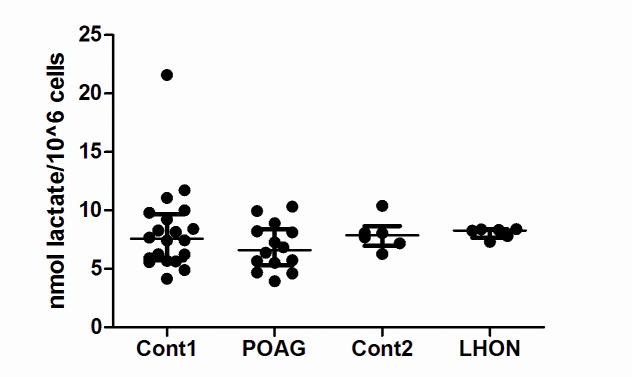
No changes in cellular lactate production in POAG or LHON lymphoblasts. Lymphoblasts were incubated in high glucose media (RPMI supplemented with glucose increased to 4mg/ml from the standard RPMI level of 2, uridine at 50 μg/ml, pyruvate at 1mM + 15% heat-inactivated FCS) for 48 hours prior to lymphoblast media supernatant being harvested for lactate measurement. There was no significant difference in extracellular lactate levels (reflective of cellular lactate production) between POAG lymphoblasts and controls, or between LHON lymphoblasts and controls. Data is median (IQR), n = 20 cont1, n = 15 POAG, n = 6 cont2 and n = 6 LHON.

As an output for detecting severe mitochondrial impairments, lymphoblasts were cultured in high glucose media for 48 hours and lactate levels were measured in the culture media. When we measured lactate levels we did not detect any significant increases in lactate levels in either LHON or POAG lymphoblasts (Fig 5). Lactate levels in POAG lymphoblasts [median (IQR): 6.6 (5.3–8.4) nmol lactate/10^6^ cells] was at similar levels to age-matched controls [median (IQR): 7.6 (5.7–9.7) nmol lactate/10^6^ cells; Mann-Whitney test, p = .25; Fig 5]. Lactate levels in LHON lymphoblasts [median (IQR): 8.3 (7.7–8.4) nmol lactate/10^6^ cells] was at similar levels to age-matched controls [median (IQR): 8.1 (7.2–9.4) nmol lactate/10^6^ cells; Mann-Whitney test, p = .63; Fig 5].

## Discussion

These data demonstrate a complex-I defect in a cohort of advanced POAG and LHON patients. This confirms our initial findings of impaired complex-I ATP synthesis in POAG [24] in a second independent cohort of advanced POAG patients using the same lymphoblast model and methods. We extend our initial findings of impaired complex-I ATP synthesis [24] to include impaired complex-I specific activity and slower proliferation in galactose media of POAG patient lymphoblasts. The degree of mitochondrial impairment in LHON patient lymphoblasts was generally greater than that found in POAG lymphoblasts. Lascaratos *et al* (2015) found that patients experiencing increased IOP but not suffering optic nerve damage (ocular hypertension) had more efficient mitochondria than age-matched controls, implying mitochondrial efficiency may predict the degree of optic nerve loss in glaucoma. We found the degree of mitochondrial impairment in advanced POAG was milder than LHON, which may be reflective of slower rates of optic nerve loss in POAG patients compared to the rapid optic nerve loss in LHON. Furthermore, enhancing mitochondrial function could preserve vision in ADOA [13] and LHON patients [44], and mitochondria are potential targets for therapeutic development [45].

The advanced POAG patients and controls were selected from the ANZRAG cohort, which has been well-characterised genetically [26–31]. A sub-group of Caucasian advanced POAG patients was selected by two ophthalmologists and clinical geneticists based on detailed clinical parameters, and matched to non-POAG controls based on ethnicity, age and gender [27]. The LHON patients all carried the 11778 G>A mutation as confirmed by Sanger sequencing, had advanced vision loss and were age-and gender matched to non-mutation carrying controls. Access to primary patient tissue (retina and optic nerve head) for glaucoma research is limited to post-mortem biopsies, however diagnosis can be unclear, and tissue samples are small which limits analysis. Modelling using peripheral tissues (e.g. transformed lymphocytes, muscle biopsies, fibroblasts) gives valuable insights into the underlying pathogenesis of mitochondrial disorders [46,47] and the lymphoblast model has been used extensively [13,24,34,47–51]. We speculate that the partial OXPHOS defects in some POAG patients contributes to energetic crisis in RGCs when combined with other stressors such as age or increased IOP. Future POAG modelling studies would benefit from more specific disease modelling from the very recent advances allowing the generation of mature retinal-ganglion cells [52] by derivation of induced pluripotent stem cells (iPSC) from primary patient fibroblasts [53].

POAG likely represents a common phenotype resulting from a number of underlying pathophysiologies, with well-recognised risk factors [54,55]. An increased inheritance of POAG among first-degree relatives [56–58] has prompted many large genome-wide association studies (GWAS) which to date have only had moderate success in identifying genes associated with rare cases of glaucoma. Analysis of POAG families with a Mendelian inheritance pattern has identified mutations in multiple loci of Optineurin (*OPTN*) [59], Myocilin (*MYOC*) [60,61] and WD repeat domain 36 (*WDR36)* [61] and copy number variation in Tank-binding kinase 1 (*TBK1*) [62]. Several GWAS studies have identified new susceptibility loci for POAG in ATP-binding cassette, sub-family A (*ABCA1)*, actin filament associated protein 1 (*AFAP1)*, GDP-mannose 4,6-dehydratase (*GMDS)* [63], phosphomannomutase 2 (*PMM2)* [64], fibronectin type III domain containing 3B (*FNDC3B)*, *rs747782* [65], SIX homeobox 6 (*SIX6)* [66], transmembrane and coiled-coil domains 1 (*TMCO1)* and CDKN2B antisense RNA 1 (*CDKN2B-AS1)* [29], and some of these genes are associated with mitochondrial function. Impaired mitochondrial function may negatively impact on the ATP-dependent cholesterol efflux by ABCA1 protein [67] and alter autophagy turnover efficiency of mitochondria by Optineurin protein [68]. Mutations in Myocilin protein reduce endogenous ATP levels, cause mitochondrial depolarisation [69] and alter a cell’s responsiveness to oxidative stress [70]. Although some of these susceptibility loci alter mitochondrial function, they still only explain a small proportion of POAG cases. An increased maternal inheritance in POAG suggests an involvement of mitochondrial genes [56,57,71–73]. Indeed, mitochondrial complex-I abnormalities have been reported for POAG [22,24] and we have confirmed this finding in a second, distinct cohort. Recent evidence suggests that variants in mitochondrial complex-I genes in POAG [22,74,75] may underlie the complex-I failure. Future genetic analyses of mitochondrial genes might expose a mitochondrial endophenotype of POAG and these studies would benefit from a combined genetic, functional and metabolomic approach.

Although GWAS studies have identified significant genes involved in glaucoma pathogenesis, the stringent statistical requirements imposed only reveal variants with the largest effects [76]. Genes or variants that individually might not reach significance, but in aggregate could be associated with a disease are often missed in GWAS. Pathway analysis of single-allele GWAS data by hypothesis-independent pathway analysis from the NEIGHBOUR and GLAUGEN datasets [77] revealed that genes in the butathione pathway, responsible for acetyl CoA metabolism, were significantly associated with POAG [78]. The current study identified that in addition to complex-I defects [24] a complex-II ATP synthesis defect was also present. Acetyl CoA is an essential Krebs cycle molecule responsible for supplying both NADH to complex-I and succinate to complex II, and any impairments in acetyl-CoA metabolism would in turn limit both complex-I and -II OXPHOS-driven ATP synthesis. Impairments in Acetyl CoA metabolism [78] likely add to the complexity of POAG pathogenesis, in that mitochondrial defects in addition to complex-I [24] are worth further investigating in POAG. In addition, only advanced POAG patients were studied in this cohort, and both a complex-I and–II ATP synthesis impairment was identified. Our first cohort examined a spectrum of mild- to severely affected POAG patients and we demonstrated a complex-I ATP synthesis defect [24]. Together this further adds to the hypothesis that the degree of mitochondrial impairment predicts POAG severity [24,25].

In summary POAG lymphoblasts demonstrated impaired complex-I specific activity and complex-I and–II ATP synthesis. When compared to LHON, the defects were less marked in POAG. The mitochondrial defect was further revealed when both POAG and LHON lymphoblasts were forced to rely on OXPHOS in galactose media where impaired lymphoblast proliferation was observed. These findings of mitochondrial impairments in POAG patients replicate our previous results from an independent cohort [24]. In all aspects the functional defects in LHON were more severe than that of POAG lymphoblasts, suggesting that a sub-group of POAG has a mitochondrial aetiology. If verified in further studies this could redirect therapeutic development and management of this common disease of ageing.

## Abbreviations

ADP: Adenosine Diphosphate
ATP: Adenosine triphosphate
ADOA: Autosomal Dominant Optic Atrophy
ANZRAG: Australian and New Zealand Registry of Advanced Glaucoma
CCCP: Carbonyl cyanide 3-chlorophenylhydrazone
DB: decylubiquinone
DBH2: decylubiquinol
EBV: Epstein Barr Virus
LHON: Leber Hereditary Optic Neuropathy
mtDNA: Mitochondrial DNA
NADH: Nicotinamide adenine dinucleotide
OXPHOS: Oxidative Phosphorylation
POAG: Primary Open-Angle Glaucoma
